# Comparison of Patient Dose and Exposure Time Across Various Techniques Used to Treat Intracranial Aneurysms: Analysis of the German Neurointerventional Database (DeGIR/DGNR) from 2018 to 2023

**DOI:** 10.1007/s00062-025-01584-7

**Published:** 2025-12-02

**Authors:** Constantin Schareck, Caroline Florack, Roland Schwab, Erelle Fuchs, Peter Schramm

**Affiliations:** 1 Institute of Radiology and Nuclear Medicine, University Hospital Schleswig-Holstein, Lübeck, Germany; 2 Institute of Neuroradiology, University Hospital Schleswig-Holstein, Lübeck, Germany; 3 Institute of Interventional Radiology, University Hospital Schleswig-Holstein, Lübeck, Germany; 4 University Clinic for Neuroradiology, University Hospital Magdeburg, Magdeburg, Germany; 5 Research Campus STIMULATE, Otto-von-Guericke University Magdeburg, Magdeburg, Germany

**Keywords:** Intracranial aneurysm therapy, Radiation protection, Intracranial intervention, Flow diverter, Coil, Stent

## Abstract

**Purpose:**

The national diagnostic reference level (DRL) for intracranial aneurysm therapy is set at 20,000 cGy cm^2^ for the patient. An adjustment between the expected dosage and exposure time requirement among a variety of applicable techniques is not commonly provided. Therefore patient dose and exposure times were analyzed when using the following techinques; dosimetric data of coils (CE), flow diverter (FD), stent-assisted coiling (SAC), intra-aneurysmatic flow diverter (IaFD), balloon-assisted coiling (BAC), flow diverter assisted coiling (FDAC), IaFD-assisted coiling (IaFDAC), x, y or t‑remodeling stent-assisted coiling (xyt-SAC), and stent-assisted IaFD (SAIaFD).

**Methods:**

A retrospective analysis of the German DeGIR-QS registry was conducted between 2018 and 2023. This study aimed to investigate the median dose-area product and exposure time for each technique, aneurysm location, type and size. The goal was to ascertain which technique is the most time- and dose-efficient.

**Results:**

FD (84.13%/79.41%) and IaFD (89.64%/73.53%) exhibited superior performance in terms of both effectiveness and efficiency when compared to CE (100% dose-area product/100% exposure time). However, certain aneurysm types and location combinations resulted in lower dose-area product and exposure time when CE was used rather than IaFD or FD. A comparison of the median dose-area product technique with the DRL reveals that all of the aforementioned procedures fall below this value. Aneurysm size has little impact on the finding that FD and IaFD are more efficient in terms of dose and time consumption than CE.

**Conclusion:**

Patients’ exposure can be reduced by selecting a technique associated with lower exposure levels, depending on the aneurysm’s location and type. However, conformity is provided for all techniques mentioned.

**Supplementary Information:**

The online version of this article (10.1007/s00062-025-01584-7) contains supplementary material, which is available to authorized users.

## Introduction

Endovascular therapy of intracranial aneurysms is a well-established and prevalent procedure in the domain of interventional neuroradiology. This assumption is supported by the consensus that the outcomes of endovascular interventions are not inferior to those of conventional neurosurgical therapies, such as microclipping, as indicated by the findings of the International Subarachnoid Aneurysm Trial (ISAT) [2, 3]. Recent studies have demonstrated that the majority of intracranial aneurysms are treated endovascularly [4]. Given the continuous advancements in technology related to various embolization therapies, it is imperative to report the radiation exposure for patients and staff in regards to the specific techniques implemented. The Federal Ministry for Radiation Protection (BfS) has explicitly published dose reference values for endovascular treatment of intracranial aneurysms, emphasizing the importance of this subject [4]. In this survey, the reference dose was not explicitly determined based on the embolization techniques, such as CE, IaFD, FD and other intrasaccular devices [5–10]. A reference value was provided, reflecting a composite of the average of the techniques utilized, and was primarily based on dose data from aneurysm embolizations with platinum coils [11–15]. The fundamental objective of all embolization techniques that are currently available and approved for the treatment of intracranial aneurysms is the elimination of the risk of bleeding associated with a cerebral artery aneurysm. A statement regarding the anticipated radiation exposure and exposure time requirements for treatments employing diverse techniques has yet to be formulated using data acquired from more than monocentric studies [16, 17]. In this context, the DeGIR-QS registry was utilized to differentiate all intracranial aneurysm embolizations that took place between the years 2018–2023. The DeGIR-QS registry is the largest database of radiological interventions in Germany. The embolization techniques used were the basis for the differentiation of the aforementioned embolizations. Subsequently, an analysis of the previously mentioned differentiations was conducted through the utilization of statistical methodologies. This approach was executed with the objective of deriving an overview of the relative radiation doses and fluoroscopy times in juxtaposition. The provided data may prove useful for interventionalists as they deliberate on the most suitable technique for the ALARA (as low as reasonably achievable) principle in the context of contemporary endovascular treatment of intracranial aneurysms of varying locations and types.

## Material and Methods

### DeGIR/DGNR Registry Data

The German Society for Interventional Radiology and Minimally Invasive Therapy (DeGIR) serves as the primary quality assurance entity for all forms of radiological interventions. Institutions can voluntarily participate in the documentation of quality assurance information. However, it is mandatory for institutions seeking DeGIR/DGNR certification or recognition as an interdisciplinary vascular center. The data subset “intracranial aneurysm embolization” from module F of the DeGIR/DGNR registry data was acquired for the years 2018 to 2023. A retrospective analysis of the data was conducted to assess the efficacy of dose-area product and exposure time in relation to the utilized technique, aneurysm location and type, as well as its dimensions. Therefore, a selection of the clinical parameters embedded within the registry data were used for the purpose of differentiation and analysis (see Table 1).

**Table 1:** Clinical parameters used for differentiation and analysis of the registry data.

DEGIR/DGNR ENTRIES		Main Data	Localization and type differentiated Data	Aneurysm size differentiated Data
**USED MATERIAL** **DOSE-AREA PRODUCT** **EXPOSURE TIME** **ANEURYSM LOCATION**		X	X	X
		X	X	X
		X	X	X
		–	X	X
**ANEURYSM TYPE**	*Fusiform/Dissected*	–	X	X
	*Saccular*	–	X	X
	*Inflammable*	–	Not enough data	Not enough data
**ANEURYSM SIZE**	*1–7* *mm*	–	–	X
	*8–15* *mm*	–	–	X
	*16–24* *mm*	–	–	X
	*≥* *25* *mm*	–	–	X

Prior to analysis, the data underwent a filtration process to eliminate any faulty data. Errors have been known to occur during the entry of study information via a web portal (Samedi GmbH, Berlin, Germany) that utilizes free text fields for input. Given that these entries lack validation through plausibility, the necessity for data filtration becomes evident. Therefore, a Python-based analysis tool with a filtration algorithm was developed. This analysis tool was created using Python along with the libraries Pandas, NumPy, and Pyplot. The filtration process is consistent with the flowchart proposed by Bärenfänger [1], with the exception that the final filtration step was excluded. In this step of the analysis, records with an absolute dose-area product value that exceeded the absolute exposure time value were excluded from further consideration. It is hypothesized that elevated dose-area product values concomitant with diminished exposure time are associated with cases where series or digital subtraction angiography (DSA) were predominantly obtained, whereas fluoroscopic acquisitions were infrequent or absent. The utilization of stents exclusively was also considered to be a factor in the rejection of records. The underlying rationale for this decision is that aneurysms cannot be effectively embolized through the mere insertion of a stent into the vessel. The utilization of these approaches is exclusively limited to the palliative treatment of very large aneurysms. In such cases, complete embolization is rendered essentially unfeasible. Consequently, the viability of a curative approach becomes a non-viable proposition.

Whereas certain groups, for example Lodi et al., implement stents in a first setting prior to aneurysm repair during a multi-stage approach, the DeGir registry data does not differentiate between single or multi-stage approaches [18]. Consequently, it can be hypothesized that single- and multi-stage stent assisted techniques were consolidated. As a result, 333 only stent-based aneurysm treatment datasets were rejected.

**Fig. 1:**
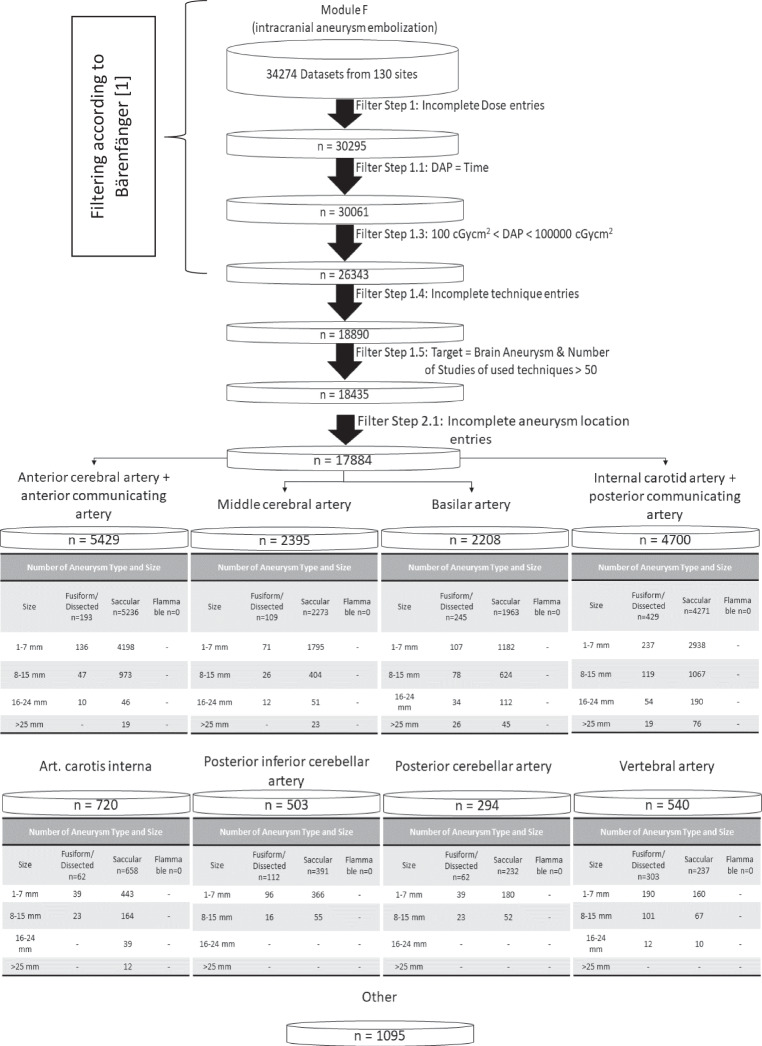
Visualization of the filtering process. Originally, 34,274 records from 130 sites were reported for the years begin 2018- begin 2023. After filtering this dataset for erroneous and incomplete data, 17,884 records remained for analysis. These records were then categorized according to the 8 most common vessels for the occurrence of aneurysms differentiated by aneurysm size and type. In this way, 1095 records were excluded from the analysis as they had a wide variety of vessel localizations and were therefore statistically insignificant. The resulting partitions were then differentiated by aneurysm size and subsequently by aneurysm type. Populations with a number lower than 10 were excluded from further procession

Following the filtration of the data, 17,884 records remained for analysis. The records were subsequently subjected to a three-pronged approach of differentiation, with the initial distinction being based on location, followed by type, and finally by size. The efficacy of each differentiation step was evaluated based on the number of remaining records. Populations with a number less than 10 were excluded from further processing. Therefore, inflammatory aneurysm types were excluded during the differentiation by aneurysm size (see Fig. 1).

## Data Evaluation

An overall comparison of the relative dose-area product and exposure time values of the 9 most commonly used techniques in relation to the dose-area product and exposure time values of coils was performed based on the filtered 17,884 data sets. These data sets were also used to obtain an overall impression of the techniques’ dose levels, i.e. the median (50th percentile) [19] for dose-area product and exposure time. This was achieved through a comparative analysis of the respective values with those of the national dose reference value of 20,000 cGycm^2^, established for intracranial aneurysm therapies (see Table 1 column Main Data). This approach was also applied to the further differentiated data subsets such as the partition with data differentiated by aneurysm location and type (see Table 1 column Localization and type differentiated Data). Finally, the median was calculated for each technique individually, as well as for each aneurysm location, type, and size. These values were then compared with the overall median dose-area product and exposure time in such aneurysm therapies. According to Vañó et al., the 75th percentile value of the distribution of median values is a salient component in the definition of DRLs. Ultimatley, an investigation was conducted to ascertain which technique yielded optimized dose values and which yielded dose values above the national diagnostic reference level [19].

The Kruskal-Wallis test was used to evaluate more than two independent data subsets for which the data does not follow a normal distribution. The null hypothesis was that there is no discrepancy between the data subsets that had been tested [20]. In this particular case, one data subset can be regarded as the median dose-area product or exposure time values of the records that have been filtered for aneurysm localization and size. This particular test is used to determine whether or not there is a statistically significant difference between the medians of the data subsets. Consequently, the number of records was also taken into consideration. The statistical significance level was set at *p* < 0.05.

For statistically significant results a post-hoc Dunn’s test was conducted. This test gives insight to how the groups, i.e. data subsets, differ exactly. This test compares pairwise the given groups and returns which of them are statistically significant. This test was chosen as a post-hoc evaluation as the test is non-parametric and does not assume the input data comes from a particular distribution. The null hypothesis was that there was no difference between the compared groups [21]. The significance value for the Dunn’s-test was set to 0.05. The statistical analyses and the test for the distribution of the data subsets (D’Agostino and Pearson’s test [22, 23]) were performed using the Python library scipy.stats.

## Results

**Fig. 2:**
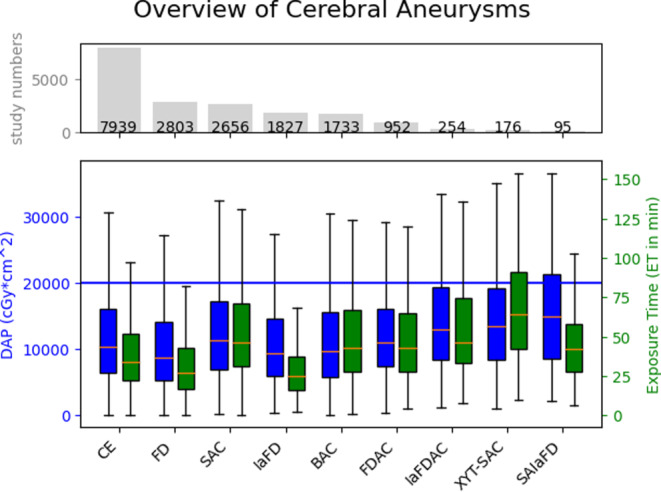
Juxtaposition of the 9 most used techniques for the treatment of intracranial aneurysms. On top of the plot the absolute recordnumber for each technique is shown. CE approaches are well suited aneurysm therapies with low radiation exposure as well as a relatively low time cost by means of the overall juxtaposition of the median values regarding DAP and ET. Only FD and IaFD fell below theses values. All other techniques surpass at least one of the two values. Anyhow, all techniques listed fell below due national diagnostic reference level (blue horizontal lien; 20,000 cGycm^2^). It can be seen that only SAIaFD based approaches surpass 20,000 cGycm^2^ in its 75th percentile

Following the filtration process, 17,448 out of 34,274 records remained for further analysis. The initial step involved the formulation of an overall expression in terms of record number, dose-area product, and exposure time. Our findings, *p* = 1.69·10^−56^ indicate that coil embolizations (CE) remain the most commonly utilized procedure; however, they are not the most efficient in terms of dosage and duration. In comparison to the CE-baseline with regard to dose-area product and exposure time, the use of a flow diverter (FD) and an intraaneurysmatic flow diverter (IaFD) was found to be more efficient with respect to both dose and time (see Fig. 2). Given the fact that not every technique can be considered for treatment of every vessel in respect to aneurysm type and size, the necessity for a more specific analysis of dose-area product and exposure time has become apparent. Consequently, a comparative analysis was conducted with regards to different vessel and aneurysm types and the utilized techniques as a result thereof. In the course of this analysis, techniques with a record count of less than 10 were excluded from consideration. The statistical significance could therefore not be ascertained.

## Evaluation by Treated Vessel Plus Underlying Aneurysm Type

**Fig. 3:**
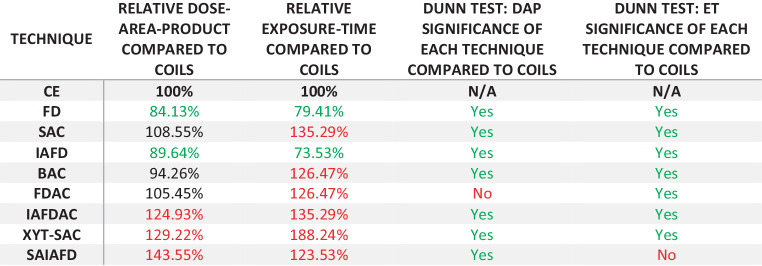
Overview of the relative values of counts, Dose-Area-Product and Exposure-Time for the filtered but yet not further differentiated regarding to aneurysm location, type and size. In this overall expression only (Ia)FD surpassed CEs’ baseline regarding to dose-area product and exposure time. All other techniques yield either similar or higher values for one or both values. Values in green: Saving potential as a percentage of CEs’ baseline value. This accounts vice versa for the red values

**Fig. 4:**
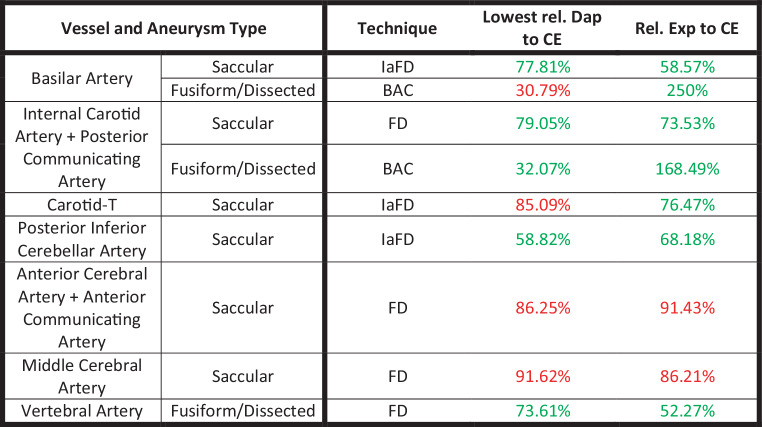
Summary of the findings for which vessel-aneurysm type combination whether technique yield highest relative dose saving potential compared to CE. The accompanied exposure time saving or increasement was also listed. Values in green indicate a significant finding. Values in red yielded an insignificant finding when compared to CE. Thus, CE can be regarded as the technique of choice in such cases

Coil embolization remains the most commonly used technique for treating intracranial aneurysms. We have shown that the dose usage and the total exposure time ranked third (see Fig. 3). Flow diverters and intra-aneurysmal flow diverters were superior to CE in this regard. However, the question arises as to whether the target vessel paired with aneurysm type influences this result in a way that changes this general finding. We therefore performed a more detailed analysis by differentiating between the same techniques for treated vessel and aneurysm type combination which were find to be overall significant. This analysis showed that the previous ranking is not universally applicable. For example, FD based therapies yield a significant dose and time reduction potential for saccular aneurysm therapies of the internal carotid artery + posterior communicating artery. In contrast the same vessel but with fusiform aneurysms showed the best dose reduction potential for BAC approaches. Similar results were discovered for treatments of the basilar artery. For saccular aneurysms, IaFD based approaches showed a dose reduction potential of 22.19% while reducing the expected exposure time by 41.43%. For fusiform associated dissected aneurysm treatments, BAC showed the lowest dose values, with a reduction down to 69.21%. However, this result appeared to be insignificant while testing the BAC-data subset against the CE-data subset for fusiform associated dissected aneurysms in the basilar artery. The same, but for different techniques, can be said about the Carotid‑T, anterior cerebral artery + anterior communicating artery and middle cerebral artery, all with saccular aneurysms (see Fig. 4). Thus, the dose reduction potential for these approaches is apparent but remains not significant compared to CE.

For a more detailed analysis, e.g. how other techniques performed in comparison to CE for the in Fig. 4 named vessel and aneurysm combinations please refer to our supplementary data.

### Evaluation According to Treated Vessel and Underlying Aneurysm Type with Consideration to Aneurysm Size and Aneurysm Rupture

**Fig. 5:**
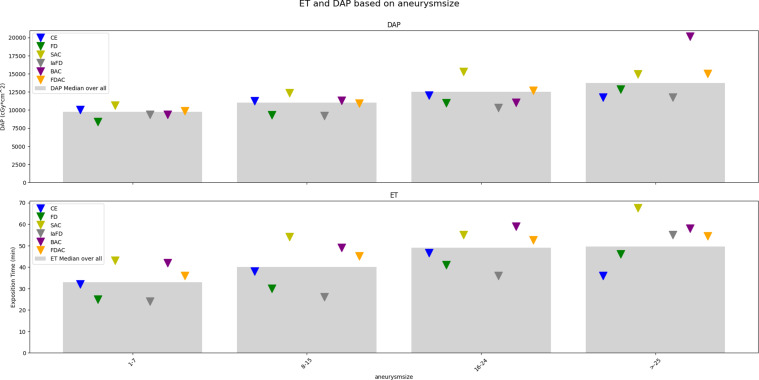
Median DAP and ET values of different techniques for embolization treatments over all vessel and aneurysm type recorded in the post filtered dataset. It is clearly to see that CE approaches reaches third place in terms of median DAP an ET. FD and IaFD clearly achieve lower values even for treatment of large aneurysms. This does not apply to aneurysm sizes greater than 25 mm as shown in this plot. This trend should be treated with high caution since the data situation became thinner and thinner with increasing sizes resulting in only a few records with high variances

The choice of technique to treat an intracranial aneurysm depends not only on the pathological vessel and aneurysm type, but also on the size of the aneurysm. Therefore, the data sets from the previous analysis were further divided into subsets according to the size of the treated aneurysm, depending on the technique used for a combination of vessel and aneurysm type. The data subsets obtained consisted of a few data sets for aneurysm sizes larger than 15 mm, where in most cases no significance was found. Therefore, no clear result could be obtained, but rather a trend of how dose-area product and exposure time values depend on the treatment of increasing aneurysm size, further categorized by the technique used (see Fig. 5).

**Fig. 6:**
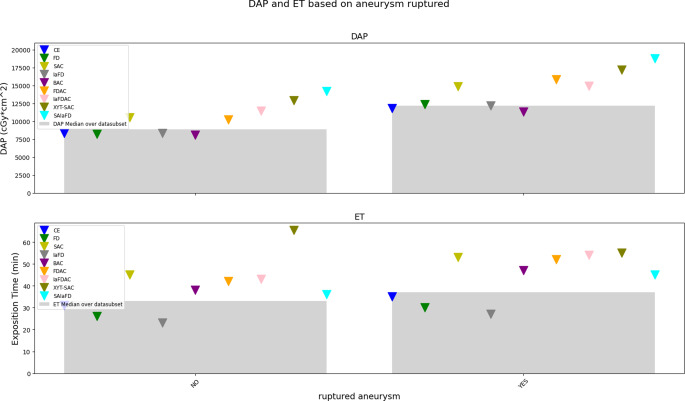
Median DAP and ET values of different techniques for embolization treatments over all vessel and aneurysm type recorded in the post filtered dataset. It is clearly to see that treatment of a ruptured aneurysm leads to an increase in DAP for each used technique. Nevertheless, ET seems to be more stable as only a small difference between ruptured and no ruptured treatment can be seen. A comparison of the dose and time consumption of the individual techniques indicates that the general statement regarding which technique results in the highest applied dose or the highest time consumption is not or negligibly small influenced by ruptured aneurysms

A further factor influencing both the dose and the exposure time is the status of the aneurysm, specifically whether it has ruptured. In order to investigate this influence, a differentiation by aneurysm type and vessel limited the significance due to the aforementioned lack of data for high differentiated data subsets. Consequently, we wanted to achieve a general impression of a trend showing how dose-area product and exposure time values are influenced by the treatment of ruptured versus non-ruptured aneurysms, in terms of the technique employed (see Fig. 6).

### Comparison to International Evaluated Dose-Reference Levels for Cerebral Aneurysm Embolization

Lopes et al. conducted a study in which published international diagnostic reference level recommendations for cerebral aneurysm embolization were compared [24]. We used this publication to obtain insight on the international feasibility of our findings. The diagnostic reference level in this study refers to the German national valid value of 20,000 cGycm^2^. Values of other countries were not tested in this study (see Table 2).

**Table 2:** Comparison of the german diagnostic reference level for intracranial aneurysm therapy with international studies regarding dose reference levels

AUTHORS	COUNTRY	Reported diagnostic reference level (cGycm^2^)	Relative Difference to 20,000 cGycm^2^^(%)^
*Kanda, R et al.* [25]	Japan	21,000	+5
*Acton, H. et al.* [26]	Ireland	12,300	−61.5
*Ihn, Y. et al.* [27]	South Korea	19,960	−0.2
*Etard, C.* [28]	France	19,000	−5
*Lee, M. et al.* [29]	USA	36,000	+80
	Switzerland	44,000	+120
	South Korea	38,350	+91.75
*Miller, D. et al.* [30]	USA	36,000	+80

It is evident that the German wide dose reference level for intracranial aneurysm therapy mirrors a comparable value to those reported in other countries. Japan, France and partial South Korea report dose reference level with a range of 19,000 to 21,000 cGycm^2^. Other studies from countries like Switzerland, USA and also South Korea, albeit inconsistently, report DRLs of more than +80% as good practice when compared to Germany. Only Ireland reported a dose reference level for intracranial aneurysm therapy lower than half of the value of the German dose reference level. Translating our findings towards this relatively low threshold we can see that every technique-vessel-aneurysm type combination would surpass this value in its 75th-percentile. The only exception were found for IaFD treatment of saccular aneurysms in the posterior inferior cerebellar artery and BAC-based treatments for fusiform respective dissected aneurysm types of the internal carotid artery and basilar artery (see Fig. S1, Fig. S2, Fig. S4).

## Discussion

Dose-area product and exposure time median values can be regarded as an expectation value of the outcome in regards to patient exposure and necessary treatment time. The selection of a particular technique is not contingent upon the physician’s level of comfort with the method.

As demonstrated in Fig. 2, the use of CE remains the predominant technique for the treatment of intracranial aneurysms. As demonstrated by the graph and its values, it can be concluded that, with regards to median dose-area product and exposure time, CE approaches are invariably positioned third, followed by FD- and lastly, IaFD-based aneurysm treatments. It is evident that a dose reduction potential exists within a range of 10.36% to 15.87%. Based on the ALARA principle (As Low As Reasonable Achievable), it might be beneficial to shift from CE techniques towards FD, and subsequently IaFD, based ones. The aforementioned statement concerns a scenario in which the treatment strategy and safety of the intervention remain largely unaltered, or, in the worst case, are not significantly compromised. The age of the patient, the location and morphology of the aneurysm are all significant factors when determining the most appropriate treatment strategy.

As demonstrated in Fig. 1, when the national diagnostic reference level is set at 20,000 cGycm^2^, it is evident that the 75th percentile of IaFDAC and XYT-SAC techniques both reach the threshold defined by this diagnostic reference level. It is evident that only SAIAFD-based approaches go over the diagnostic reference level threshold. In consideration to the findings reported by Vañó et al. [19], it can be concluded that the 75th percentile can be designated as the diagnostic reference level for a specific technique. The importance of a precise selection of technique does not only induce a reduction or increase in patient dose. The radiation exposure of the treating personnel is directly proportional to the applied patient dose, under the assumption that the position of the physician and the usage of radiation protection in the room remains constant. Therefore, the overall experienced dose of the physicians is proportionate to the employed technique and its expected dose expenditure. Consequently, a modification of techniques may enhance the radiation protection of personnel performing the intervention [31]. In addition to the general considerations regarding dosage, the reduced treatment time for FD- and IaFD in comparison to the baseline of CEs has the potential to yield further organizational and temporal benefits for the overall course of treatment. This has the potential to result in a reduction of other risks such as those associated with prolonged anaesthesia exposure [32]. It is evident that transitioning from CE to FD-IaFD techniques would result in a reduction of the required exposure time of 20.57% and 26.47%, respectively. This phenomenon can be attributed to the faster release of FD and IaFD techniques. The release of CE is indicative of a multi-device release within the aneurysm. FD and IaFD embolisations require a single device release, consequently resulting in reduced procedure times [33, 34].

As mentioned before this can be regarded as a general assessment. It is important to acknowledge the valid criticism that not all techniques are applicable to every vessel and aneurysm type. It is imperative to consider the potential risks associated with the use of FD, particularly the risk of bleeding during subsequent interventions due to the inhibition of platelet aggregation. Should the selection of technique prove to be an ineffective factor in altering the potential risks to patients, the dose reduction and time efficiency aspects described in the results section can be taken into consideration. In such cases, the selection of the technique can be made on the basis that CE techniques never attain the first position in terms of median dose-area product and exposure time. Predominant category of FD or IaFD yield a reduction potential for dose-area product and exposure time simultaneously. It has been demonstrated that alternative techniques, such as BAC, have the capacity to yield a lower dose-area product, however, at the cost of an elevated exposure time. In the case of fusiform aneurysm types, BAC has the potential for yielding highest dosage reduction, requiring approximately 30% dose-area products in comparison to CE techniques. However, it should be noted that this procedure is associated with a significant increase in exposure time of approximately 70%. It has been established that digital subtraction angiography (DSA) and serial images are associated with a higher dose when compared to fluoroscopic images. Therefore, the reduction in dose with a concurrent increase in exposure time can be elucidated by the predominant acquisition of fluoroscopic images [35]. It is reasonable to hypothesize that this choice is logical, since both coils and balloons are easily visible under fluoroscopy due to their high contrast in the image [36]. All other combinatory techniques like IaFDAC are prone to yield higher doses and exposure times. In certain instances, such as the embolisation of saccular aneurysms of the carotid‑T, such techniques have been observed to yield a lower median dose-area product in comparison to CE techniques (see Fig. S3 & Tab. S3). Nevertheless, they never fell below that of FD and IaFDs. The statistical significance in our findings therefore needs to be viewed in a slightly different way. Dunns-test showed (Fig. 3) that differentiation for just the used technique yield significant results between the different techniques for intracranial aneurysm embolization. This can be traced back to the fact that without complex differentiation of the datasets, for vessel and aneurysm type, subsets of sufficient size were available for testing. With further differentiation the size of each available dataset shrinks for testing. Thus, variance in the resulting datasets rises and consists out of only a few entries. The experience of the interventionalist performing the intervention, and the angiography systems used, are two factors that have been found to have a central role in the variance of the data set obtained. The Median as well gets more and more prone to outliers. Testing such subsets against subsets with larger populations and thus a more stable variance and median, can therefore result in no significance, yet can give an interesting insight towards patient exposition and how it is paired to the used technique. The analysis of aneurysm size for a given vessel and aneurysm-type combination, differentiated for each technique, resulted in mostly non-significant findings. The investigation was constrained in its ability to achieve a comprehensive overview, due to the inability to ascertain the influence of aneurysm size on the selection of technique, median dose-area product and exposure time for the used techniques. As would be anticipated, an increase in median dose-area product and exposure time is observable with escalating aneurysm size. However, it is evident that FD, IaFD, CE and BAC-based techniques yield median dose-area product values that are lower than the overall median dose-area product across all techniques. It can thus be concluded that the aforementioned facts are not biased by aneurysm size. IaFD and FD are applicable for all recorded aneurysm sizes and yield lower median dose-area product and exposure time values than CE techniques. However, it should be noted that the values for therapies of aneurysms larger or equal to 25 mm are obtained through analysis of datasets consisting of only a few records. Therefore, the considerable variation between the records results in median values that do not accurately reflect the trend observed in therapies for aneurysms of smaller sizes. It is important to note that the range of techniques available for the treatment of aneurysms larger than 25 mm is considerably restricted. This may provide a rationale for the observation that CE techniques are found on a lower dose-area product-level as for sizes in the range of 16 mm to 24 mm. It has been shown that the dosage and exposure time are strongly influenced by various parameters. One of the most important parameters, in addition to those already mentioned, is the state of the aneurysm, i.e. whether it is ruptured or not. The present study addresses this issue by presenting this influence on the dosage and exposure time for our listed techniques in general. It must be mentioned that no differentiation by vessel and aneurysm type was made for this task, as the analysis of whether or not the aneurysm was ruptured for a given vessel and aneurysm type combination, differentiated for each technique, yielded mostly non-significant results. A fascinating discovery was made upon analysis of the median dose and exposure time expenditure for all techniques over the total median dose. This analysis showed that the respective technique placement from one to the other was almost completely independent of the aneurysm status. A higher dosage was required, but at a similar level, for each technique. Therefore we can conclude that for the treatment of a ruptured aneurysm more series are required instead of pure fluoroscopy. This statement is strengthened by the fact that median exposition time in general and for each technique does not rise at an equal amount compared to the increase in dose area product, when it comes to ruptured aneurysm treatments.

This study shows possible justifications for exceeding the national dose reference values with respect to the different 75th percentiles of each technique dependent on a given combination of vessel and aneurysm type. A call for technique-based national dose reference values has not been made, as we have shown that dose in the treatment of intracranial aneurysms is highly influenced by more than just the choice of technique. Nevertheless, the choice of technique can improve the radiation protection of the patient and the staff in the room. These results can also be used internationally. Most of the internationally reported dose reference values for intracranial aneurysm embolization’s are in a similar range as the German dose reference value of 20,000 cGycm^2^. Sites in countries with higher dose reference values in the range of those reported by the USA or Switzerland can use this study to adjust these values in line with the ALARA principle. There is only one reported dose reference level of 12,300 cGycm^2^, which is unattainable for the 75th percentile of any technique in this study. Since this reported value is based on a local, monocentric study with only one angiography system implemented, the influence of the treated techniques, vessels, aneurysm types and sizes are highly biased and cannot be applied generally.

## Limitations

It is acknowledged that the present study was conducted on the basis of certain assumptions regarding the DeGIR registry data, as outlined in the corresponding section. As a consequence of the aforementioned reasons, one of the more prominent approaches is to discard only stent-based aneurysm repairs. It is acknowledged that some of these entries may have been first setting multi-stage approaches, which were not considered in the present study. This may also account vice versa. Therefore, for instance, second-setting multi-stage approaches could be incorporated into our CE subset, as the DeGIR entry exclusively addresses the actual aneurysm repair. Further limitations result from the lack of dose-influencing relevant data in the DeGIR records. For example, there is a lack of information on the actual experience of the interventionalist performing the procedure and which angiography system was used. It is obvious that experience on the one hand and the use of state-of-the-art angiographic systems on the other hand can have a considerable influence on the dose and the time required.

Another limitation is the lack of procedural standardization in intracranial aneurysm therapy between the varying centers that upload to the DeGIR data bases.

This study is a retrospective analysis of data recorded from 2018 to 2023. Therefor it is possible that after 2023, there have been new developed techniques for intracranial aneurysm therapy that were not included in this study. The DeGIR-entry data does not represent analytical data of a randomized clinical study but a large collection of real-world data.

For reasons of readability of the study, we have limited our data presentation of the results from the Dunns test as our main focus, i.e. the comparison of coil embolization with the 8 most used techniques. We would like to offer readers the opportunity to contact us if they are interested in data that was not included in this study.

## Conclusion

CE based techniques are most frequently used in intracranial embolisations, however, they are not the most dose-efficient. Techniques such as FD and IaFD offer reduced radiation exposure and shorter treatment times. When clinical conditions allow flexibility in technique selection without compromising patient safety or treatment success, tailoring the approach to aneurysm and vessel characteristics can improve radiation protection in line with the ALARA principle. This may also enhance procedural efficiency. While the variability in influencing factors precludes the establishment of technique-specific national dose reference values, these findings offer justification for exceeding current thresholds when appropriate. This applies not only to the German reference value of 20,000 cGy·cm^2^ but also to international benchmarks, which often fall within or above this range. Therefore, the results are broadly applicable and support a more individualized, efficiency-focused approach to intracranial aneurysm embolisations.

## Supplementary Information

The supplementary includes graphs and tables for each mentioned vessel and aneurysm combination analysed towards dosage and exposure time expenditure for the 9 most used techniques. Thus it gives a more precise overview of the data shown in Fig. 4.
