# Supplementary material for: Comparison of Patient Dose and Exposure Time Across Various Techniques Used to Treat Intracranial Aneurysms: Analysis of the German Neurointerventional Database (DeGIR/DGNR) from 2018 to 2023
Source: Clin Neuroradiol. 2025 Dec 2;36(2):439–49. doi: 10.1007/s00062-025-01584-7 (PMC13319991; doi:10.1007/s00062-025-01584-7)
Supplement: Supplementary file 1 — The supplementary includes graphs and tables for each mentioned vessel and aneurysm combination analysed towards dosage and exposure time expenditure for the 9 most used techniques. Thus it gives a more precise overview of the data shown in Fig. 4. [file 62_2025_1584_MOESM1_ESM.docx]

**Evaluation by treated vessel plus underlying aneurism type**


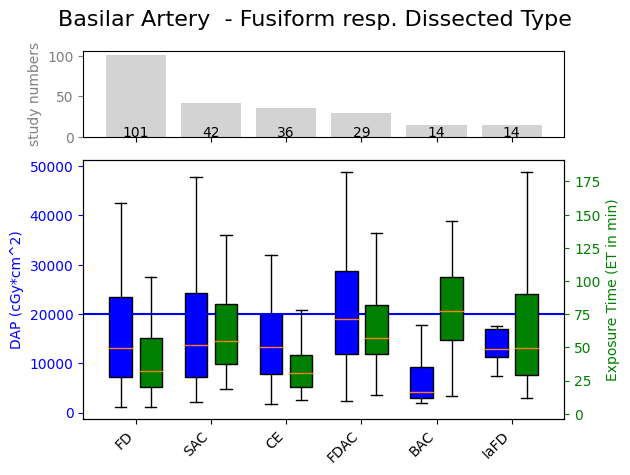
**
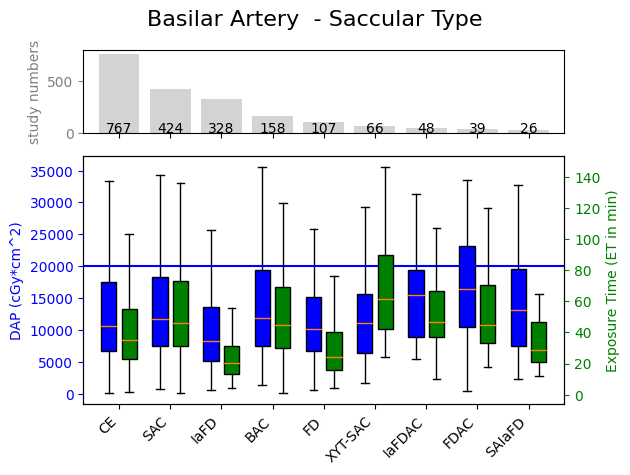
Basilar artery with saccular and fusiform aneurysm types**

Fig.S1: Juxtaposition graphs of embolization techniques for treatment of saccular and fusiform aneurysms of the basilar artery. The absolute record number is shown on top of each of the graphs. The big blue horizontal line indicates the national diagnostic reference level of 20,000 cGycm^2^.

CE-based approaches remain the most prevalent technique, with 39.07% of records indicating its use in the treatment of a saccular aneurysm of the basilar artery. This approach is analogous to 9.66% of the overall coil-based embolization methodologies. It is noteworthy that exclusively IaFD-based approaches demonstrate superior efficiency in terms of dose-area product (77.81%) and exposure time (58.75%). In contrast, FD yielded a reduction in exposure time equivalent to 68.57%, yet without a concomitant significant decrease in exposure, which was 95.97%. In the case of the treatment of fusiform aneurysms, both techniques demonstrate a tendency to approach the median dose-area product of coil-based embolizations. Furthermore, the implementation of IaFD-based approaches has been shown to result in an increase in exposure time by 59.68%. For this vessel and aneurysm type combination, the use of BAC techniques results in a 69.21% reduction in exposure. However, these techniques also yield the highest exposure times, with an increase of 150% compared to CE approaches (see Fig.S1 & Tab.S1). The significance for this analysis was p_saccular_ = 7.15·^-13^ and p_fusiform_ = 0.007 for dose-area product, p_saccular_ = 4.19·10^-69^ and p_fusiform_ = 3.09·^-6^ for exposure time.

| Technique | Relative Dose-Area Product Compared to Coils | Relative Exposure Time Compared to Coils | Dunn test: DAP significance of each technique compared to Coils | Dunn test: ET significance of each technique compared to Coils | |
| --- | --- | --- | --- | --- | --- |
|  | Basilar artery with saccular aneurysm  n=1963 | | | |  |
| CE | 100% | 100% | N/A | N/A | |
| FD | 95.97% | 68.57% | No | Yes | |
| SAC | 110.68% | 131.43 | No | Yes | |
| IaFD | 77.81% | 58.57% | Yes | Yes | |
| BAC | 111.58% | 128.57% | No | Yes | |
| FDAC | 154.98% | 128.57% | Yes | No | |
| IAFDAC | 145.60% | 134.29% | No | Yes | |
| xyt-SAC | 104.82% | 175.71% | No | Yes | |
| SAIaFD | 124.29% | 82.86% | No | No | |
|  | Basilar artery with fusiform aneurysm  n=236 | | | |  |
| CE | 100% | 100% | N/A | N/A | |
| FD | 98.42% | 103.23% | No | No | |
| SAC | 103.71% | 176.13% | No | Yes | |
| IaFD | 98.01% | 159.68% | No | No | |
| BAC | 30.79% | 250% | No | Yes | |
| FDAC | 142.77% | 183.87% | No | Yes | |

Tab.S1: Relative values of the used techniques for embolization treatment of the basilar artery with either saccular or fusiform aneurysm types. Values in green: Saving potential as a percentage of CEs’ baseline value. This accounts vice versa for the red values.

**Internal carotid artery + posterior communicating artery** **with saccular and fusiform aneurysm types**

^
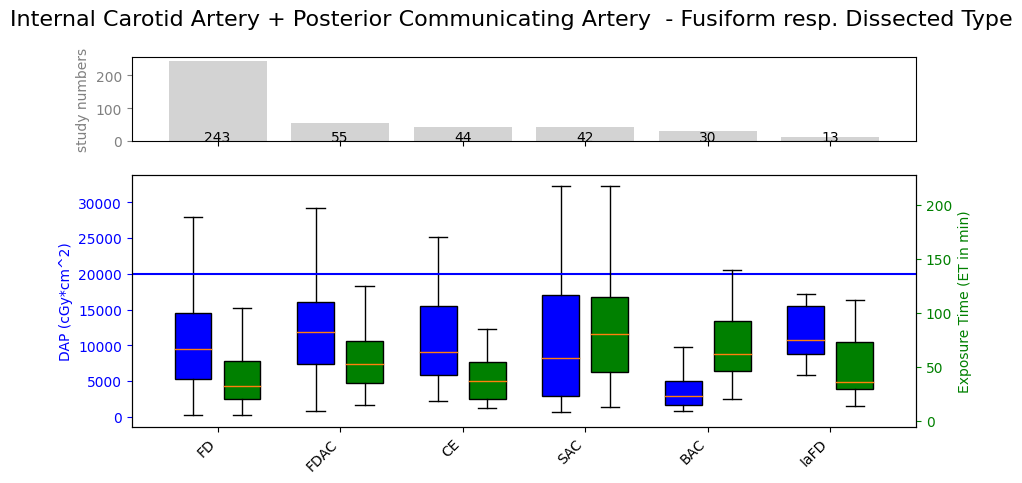

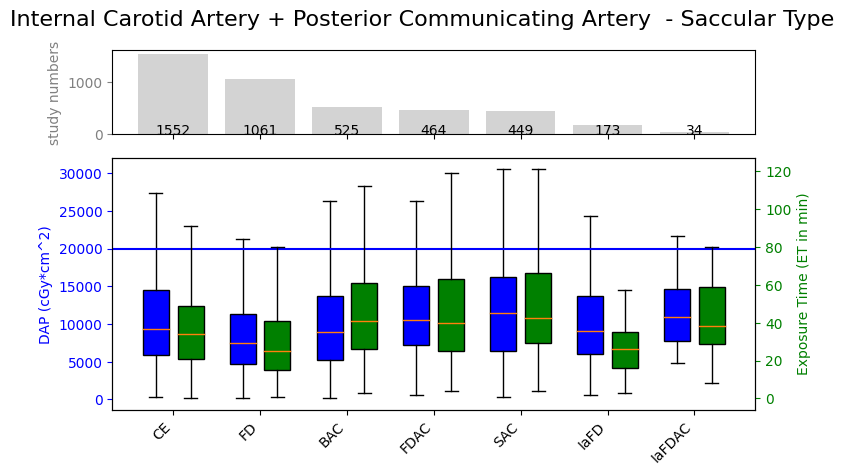
^ CE approaches remains the most commonly used technique, with 36.45% of the records involving the treatment of a saccular aneurysm of the internal carotid artery as well as the posterior communicating artery (ACI + PCOM). This is equivalent to 19.55% of the overall CE approaches. It is noteworthy that FD-based approaches have been shown to yield higher efficiency in terms of dose-area product (79.05%) and exposure time (73.53%). In contrast, IaFD yielded a reduction in exposure time equivalent to 76.47%, yet without a significant decrease in dose-area product (96.65%).
In the case of the treatment of a fusiform aneurysm, both techniques yielded higher dose-area product compared to CE. While IaFD embolizations was found to be 17.84% higher than the median

Fig.S2: Juxtaposition graphs of embolization techniques for treatment of saccular and fusiform aneurysms of the internal carotid artery + posterior communicating artery. The absolute record number is shown on top of each of the graphs. The big blue horizontal line indicates the national diagnostic reference level of 20,000 cGycm^2^.

dose-area product, the exposure time nears CE’s baseline. It has emerged that FD-based approaches yield a slight benefit in terms of exposure time, with an observed percentage of 87.67%, in comparison to the CE's baseline. Nevertheless, FD-based approaches seem to be the technique of choice, since 56.91% of all treatments of this data subset were performed using this technique. For the treatment of internal carotid artery and posterior communicating artery combinations with fusiform aneurysms, the BAC technique yielded the most significant reduction in exposure with 67.93% dose-area product, while simultaneously exhibiting the highest exposure times with 68.49% increase compared to CE (see Fig.S2 & Tab.S2). Each technique fell below 20,000 cGycm^2^ for both vessel and aneurysm type combinations. The significance for this analysis was set at p_saccular_ = 2.09·10^-23^ and p_fusiform_ = 1.99·10^-5^ for dose-area product, p_saccular_ = 5.95·10^-72^ and p_fusiform_ = 1.97·10^-13^ for exposure time

| Technique | Relative Dose-Area Product Compared to Coils | Relative Exposure Time Compared to Coils | Dunn test: DAP significance of each technique compared to Coils | Dunn test: ET significance of each technique compared to Coils | |
| --- | --- | --- | --- | --- | --- |
|  | Internal carotid artery + Posterior communicating artery with saccular aneurysm   n = 4258 | | | | |
| CE | 100% | 100% | N/A | | N/A |
| FD | 79.05% | 73.53% | Yes | | Yes |
| SAC | 121.91% | 125.21% | Yes | | Yes |
| IaFD | 96.65% | 76.47% | No | | Yes |
| BAC | 95.35% | 120.59% | No | | Yes |
| FDAC | 111.75% | 117.65% | Yes | | Yes |
| IAFDAC | 116.10% | 111.76% | No | | No |
|  | Internal carotid artery + Posterior communicating artery with fusiform aneurysm  n = 427 | | | | |
| CE | 100% | 100% | N/A | | N/A |
| FD | 103.71% | 87.67% | No | | No |
| SAC | 90.07% | 220.55% | No | | Yes |
| IaFD | 117.84% | 98.63% | No | | No |
| BAC | 32.07% | 168.49% | Yes | | Yes |
| FDAC | 130.42% | 145.21% | No | | No |

Tab.S2: Relative values of the used techniques for embolization treatment of the internal carotid artery plus posterior communicating artery with either saccular or fusiform aneurysm types. Values in green: Saving potential as a percentage of CEs’ baseline value. This accounts vice versa for the red values.

| Technique | Relative Dose-Area Product Compared to Coils | Relative Exposure Time Compared to Coils | Dunn test: DAP significance of each technique compared to Coils | Dunn test: ET significance of each technique compared to Coils |
| --- | --- | --- | --- | --- |
|  | Carotid-T with saccular aneurysm  n = 639 | | | |
| Coils | 100.00% | 100.00% | N/A | N/A |
| FD | 88.01% | 85.29% | No | No |
| SAC | 121.11% | 150.00% | No | Yes |
| IaFD | 85.09% | 76.47% | No | Yes |
| BAC | 89.12% | 107.35% | No | No |
| FDAC | 127.84% | 129.41% | No | No |

**Carotit-T with saccular aneurysm type**

Tab.S3: Relative values of the used techniques for embolization treatment of the carotid-T with only saccular aneurysm types. Values in green: Saving potential as a percentage of coils’ baseline value. This accounts vice versa for the red values.

**
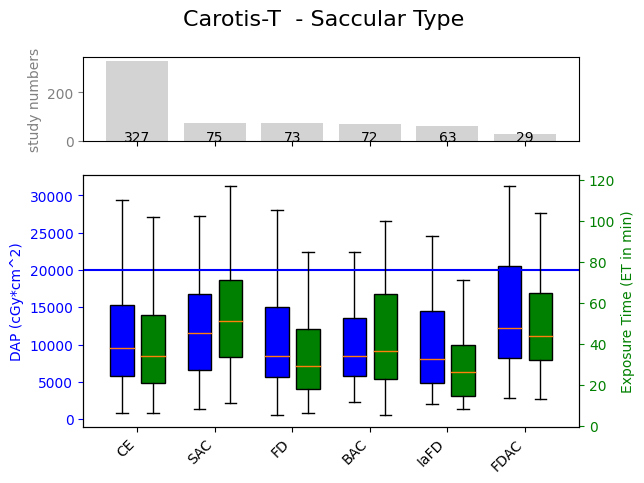
** CE continues to be the most commonly employed technique with 51.17% of records for the treatment of a saccular aneurysm of the carotit terminus. This represents 4.11% of the total CE procedures. SAC (11.74%), FD (11.42%), BAC (11.27%), and IaFD (9.86%) based embolization approaches were used relatively equally frequently. However, SAC is considered the second most commonly used technique. Nevertheless, its median dose-area product and exposure time values exceeded the CE baseline by 21.11% and 50%, respectively. All other techniques used, except FDAC-based approaches, were below these values in terms of median DAP. In terms of exposure time only FD (85.29%) and IaFD (76.47%) fell below, while BAC based technique approached (107.35%) and FDAC exceeded this baseline by 29.41%. The median dose-area product of all techniques used fell below the national diagnostic reference level of 20,000 cGycm^2^ (see Fig.S3 & Tab.S3). Only the 75^th^ percentile of the FDAC techniques slightly exceeds this value. The significance for this analysis was found to be p = 0.038 for dose-area product and p = 5.72·10^-8^ for exposure time. In the case of fusiform aneurysm types, no significance could be found for this vessel, so no results are shown.

Fig.S3: Juxtaposition graph of the DAP and ET for different techniques shown in boxplots. On top of the graph the absolute record number for this specific vessel and aneurysm type can be found. The big blue line indicates the national diagnostic reference level

| Technique | Relative Dose-Area Product Compared to Coils | Relative Exposure Time Compared to Coils | Dunn test: DAP significance of each technique compared to Coils | Dunn test: DAP significance of each technique compared to Coils |
| --- | --- | --- | --- | --- |
|  | PICA with saccular aneurysm  n = 424 | | | |
| CE | 100.00% | 100.00% | N/A | N/A |
| FD | 94.65% | 69.70% | No | Yes |
| SAC | 114.67% | 127.27% | No | No |
| IaFD | 58.82% | 68.18% | Yes | Yes |
| BAC | 101.83% | 109.09% | No | No |
| FDAC | 85.58% | 146.97% | No | No |

Tab.S4: Relative values of the used techniques for embolization treatment of the arteria cerebelli posterior inferior with only saccular aneurysm types. Values in green: Saving potential as a percentage of coils’ baseline value. This accounts vice versa for the red values.

^
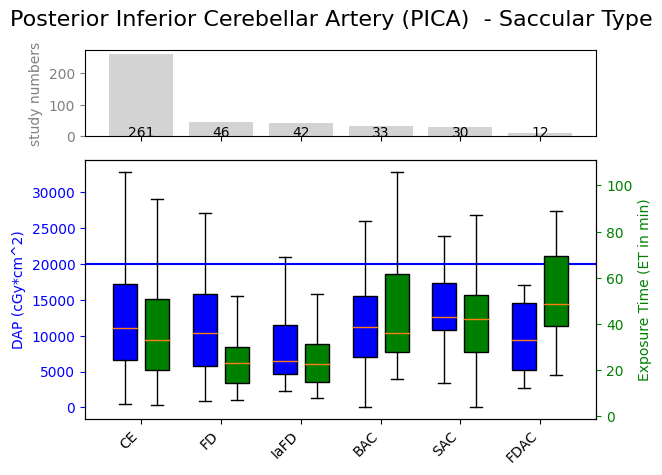
^**Posterior inferior cerebellar artery (PICA) with saccular aneurysm type**

 Looking at the records for aneurysm therapy of the posterior inferior cerebellar artery with saccular aneurysm type, we can see that the CE approach remains the most used with 61.56% of all records for this subset. This is similar to 3.29% of all remaining CE records after filtering. A median comparison shows that this technique is only in third place in terms of median dose-area product and exposure time. IaFD and FDAC based approaches yield lower median dose-area product with 58.82% and 85.58% respectively. CE therapies also rank third in terms of median exposure time. In this case, FD and IaFD techniques are more time efficient with 69.70% and 68.18% compared to CE's baseline exposure time. FD-based approaches are successful only when compared to exposure time, while dose-area product is similar to CE's baseline. The reverse is true for FDAC. IaFD are more efficient in both respects. The significance was p = 0.005 for dose-area product and p = 3.75·10^-7^ for exposure time. No significance was found for the combination of this vessel with fusiform aneurysm types (p = 0.9), so no results are listed. All of the listed techniques used to treat this type of aneurysm combination fell below the national diagnostic reference level of 20,000 cGycm^2^ (see Fig.S4 & Tab.S4).

Fig.S4: Juxtaposition graph of the DAP and ET for different techniques shown in boxplots. On top of the graph the absolute record number for this specific vessel and aneurysm type can be found. The big blue line indicates the national diagnostic reference level.


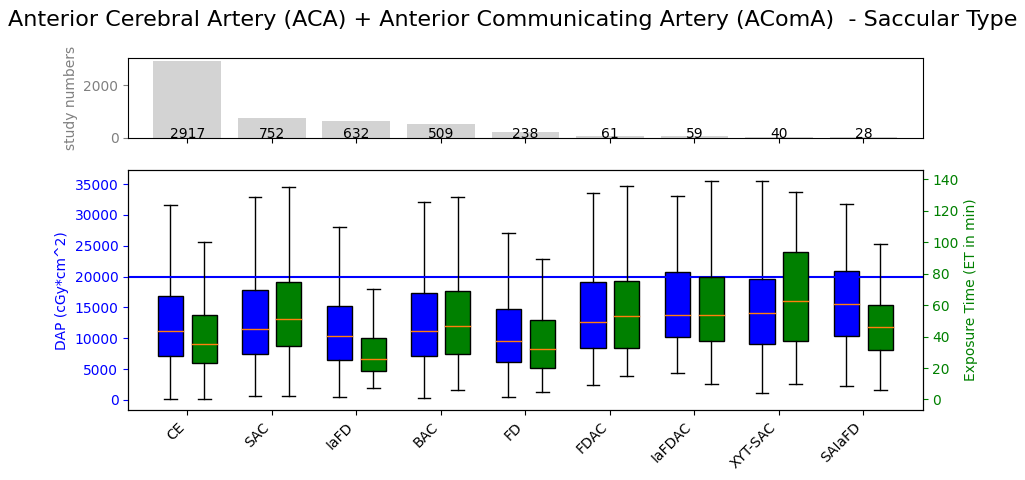
**Anterior cerebral artery (ACA) + Anterior communicating artery (AComA) with saccular aneurysm type**

Fig.S5: Juxtapositiongraph of the DAP and ET for different techniques shown in boxplots. On top of the graph the absolute record number for this specific vessel and aneurysm type can be found. The big blue line indicates the national diagnostic reference level.

Looking at the embolization datasets for the ACA including AComA, CE approaches continue to be the most commonly used technique for saccular and fusiform aneurysms with 55.71% and 37.70% employment rates, respectively. Comparing these numbers with the total number of CE-based datasets, it can be seen that embolization with coils from this dataset represents more than one third (36.74%) of all datasets. Thus, this technique might suggest the best efficiency in terms of median dose-area product and exposure time. A comparison with the other medians proves this to be false. It can be seen that only FD-based embolizations with an appearance of 8.49% give a better dose efficiency with 86.25% of the CE median dose-area product. It is also associated with a decreasing exposure time with 91.43% of CE's median exposure time. The best increase in efficiency can be found with IaFD based approaches with 74.29% of the CE's median exposure time. However, the decrease in median dose-area product for this technique remains similar to that of CE's baseline at 92.62%. All other techniques either exceed or are similar to CE's median dose-area product and exposure time. In terms of conformity, all techniques fall below the national diagnostic reference level of 20,000 cGycm2, except for the IaFDAC and SAIaFD-based approaches falling in the 75th percentile (see Fig.S5 & Tab.S5). The significance was set at p = 2.79·10^-9^ for dose-area product and p = 1.23·10^-96^ exposure time. For fusiform aneurysm type embolization of this vessel no significance with p =0.10 was found and thus no results are listed.

| Technique | Relative Dose-Area Product Compared to Coils | Relative Exposure Time Compared to Coils | Dunn test: DAP significance of each technique compared to Coils | Dunn test: ET significance of each technique compared to Coils |
| --- | --- | --- | --- | --- |
|  | ACA + AComA with saccular aneurysm  n = 5236 | | | |
| CE | 100.00% | 100.00% | N/A | N/A |
| FD | 86.25% | 91.43% | No | No |
| SAC | 103.64% | 145.71% | No | Yes |
| IAFD | 92.62% | 74.29% | No | Yes |
| BAC | 99.96% | 134.29% | No | Yes |
| FDAC | 114.08% | 151.43% | No | Yes |
| IAFDAC | 123.61% | 154.29% | Yes | Yes |
| xyt-SAC | 126.53% | 178.57% | No | Yes |
| SAIafd | 139.34% | 131.43% | No | No |

Tab.S5: Relative values of the used techniques for embolization treatment of the ACA + AComA with saccular and fusiform aneurysm types. Values in green: Saving potential as a percentage of CEs’ baseline value. This accounts vice versa for the red values.

**Middle cerebral artery (MCA) with saccular aneurysm type**

For the treatment of MCA saccular aneurysms, the CE technique ranks second in terms of median dose-area product and exposure time. However, it is again the most commonly used technique for such treatments with an overall usage of 40.21% for this data subset. This corresponds to 11.51% of all CE procedures. Only FD-based embolization techniques yield better median values in terms of dose-area product and exposure time compared to the CE baseline with 91.61% and 86.21%, respectively. IaFD only comes close in terms of time savings with a 24.14% decrease in exposure time
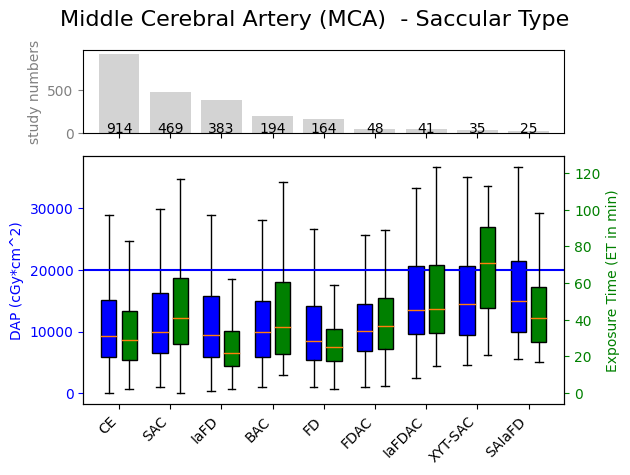
compared to the median exposure time of CEs. All other techniques result in either similar or dramatically increased median dose-area product and exposure time in comparison. The techniques IaFDAC, xyt-SAC and SAIaFD surpass the 75^th^ percentile in the national diagnostic reference level.

Fig.S6: Juxtaposition graph of the DAP and ET for different techniques shown in boxplots. On top of the graph the absolute record number for this specific vessel and aneurysm type can be found. The big blue line indicates the national diagnostic reference level.

| Technique | Relative Dose-Area Product Compared to Coils | Relative Exposure Time Compared to Coils | Dunn test: DAP significance of each technique compared to Coils | Dunn test: ET significance of each technique compared to Coils |
| --- | --- | --- | --- | --- |
|  | MCA with saccular aneurysm  n = 2273 | | | |
| CE | 100.00% | 100.00% | N/A | N/A |
| FD | 91.62% | 86.21% | No | No |
| SAC | 107.15% | 141.38% | No | Yes |
| IaFD | 101.53% | 75.86% | No | Yes |
| BAC | 105.76% | 124.14% | No | Yes |
| FDAC | 108.57% | 125.86% | No | No |
| IAFDAC | 145.03% | 158.62% | Yes | Yes |
| xyt-SAC | 155.12% | 244.83% | Yes | Yes |
| SAIAFD | 160.30% | 141.38% | Yes | No |

Tab.S6: Relative values of the used techniques for embolization treatment of the arteria cerebri media with saccular aneurysm types. Values in green: Saving potential as a percentage of CEs’ baseline value. This accounts vice versa for the red values.

The significance was found to be p =2.89·10^-6^ for dose-area product and p = 2.05·10^-52^ exposure time. No significance was found for treatment of the same vessel with fusiform aneurysm type (see Fig.S6 & Tab.S6).

**Vertebral artery (VA) with fusiform aneurysm type**


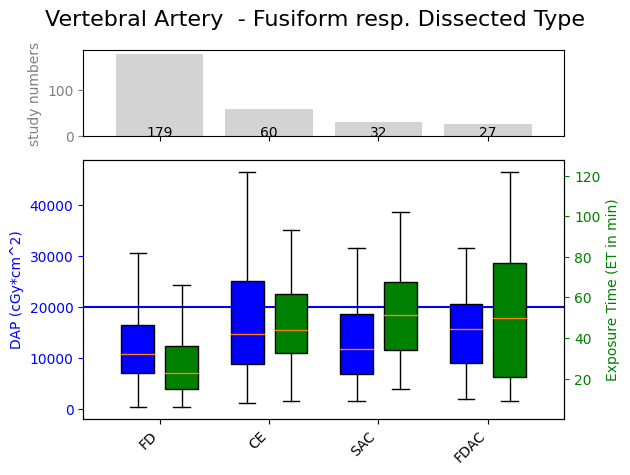
 The last significant finding with p = 0.02 for dose-area product and p = 6.19·10^-12^ for exposure time was for the data subset embolization of vertebral artery with fusiform aneurysm type. In contrast to the other data subsets mentioned above, FD was the most frequently used technique with 60.07% of such procedures. CE techniques were used in only about a third of these cases (20.13%). In addition, this technique is more likely to exceed the national diagnostic reference level of 20,000 cGycm2. Nevertheless, FD gives a higher efficiency in terms of median dose-area product and exposure time. The median dose-area product is reduced by 26.39%, while the median exposure time is reduced by 47.73%. This shows a high saving potential in terms of the time required to use this technique. Only SAC techniques also yield a dose-area product reduction of 20.84% compared to CE based approaches (see Fig.S7 & Tab.S7).

Fig.S7: Juxtaposition graph of the DAP and ET for different techniques shown in boxplots. On top of the graph the absolute record number for this specific vessel and aneurysm type can be found. The big blue line indicates the national diagnostic reference level.

| Technique | Relative Dose-Area Product Compared to Coils | Relative Exposure Time Compared to Coils | Dunn test: DAP significance of each technique compared to Coils | Dunn test: ET significance of each technique compared to Coils |
| --- | --- | --- | --- | --- |
|  | Vertebral artery with fusiform aneurysm  n = 298 | | | |
| CE | 100.00% | 100.00% | N/A | N/A |
| FD | 73.61% | 52.27% | Yes | Yes |
| SAC | 79.16% | 117.05% | No | No |
| FDAC | 106.64% | 113.64% | No | No |

Tab.S7: Relative values of the used techniques for embolization treatment of the Vertebral artery with saccular and fusiform aneurysm types. Values in green: Saving potential as a percentage of CEs’ baseline value. This accounts vice versa for the red values.
